# A Molecular Method to Discriminate between Mass-Reared Sterile and Wild Tsetse Flies during Eradication Programmes That Have a Sterile Insect Technique Component

**DOI:** 10.1371/journal.pntd.0004491

**Published:** 2016-02-22

**Authors:** Soumaïla Pagabeleguem, Geoffrey Gimonneau, Momar Talla Seck, Marc J. B. Vreysen, Baba Sall, Jean-Baptiste Rayaissé, Issa Sidibé, Jérémy Bouyer, Sophie Ravel

**Affiliations:** 1 Pan-African Tsetse and Trypanosomiasis Eradication Campaign, Bobo-Dioulasso, Burkina Faso; 2 CIRAD, UMR CMAEE, Montpellier, France; 3 Institut Sénégalais de Recherches Agricoles, Laboratoire National d’Elevage et de Recherches Vétérinaires, Service de Bio-écologie et Pathologies Parasitaires, Dakar - Hann, Sénégal; 4 CIRAD, UMR INTERTRYP, Montpellier, France; 5 Centre International de Recherche-Développement sur l’Élevage en Zone Subhumide, Bobo-Dioulasso, Burkina Faso; 6 Insect Pest Control Laboratory, Joint FAO/IAEA Programme of Nuclear Techniques in Food and Agriculture, International Atomic Energy Agency, Vienna, Austria; 7 Direction des Services Vétérinaires, Dakar, Sénégal; 8 National Institute for Tsetse and Trypanosomosis Control and Eradication, Livestock Development Sector, Ministry of Agriculture, Addis Ababa, Ethiopia; 9 Pan-African Tsetse and Trypanosomiasis Eradication Campaign Coordination Office, Rural Economy and Agriculture Department, African Union Commission, Addis Ababa, Ethiopia; 10 IRD, UMR INTERTRYP, Montpellier, France; IRD/CIRDES, FRANCE

## Abstract

**Background:**

The Government of Senegal has embarked several years ago on a project that aims to eradicate *Glossina palpalis gambiensis* from the Niayes area. The removal of the animal trypanosomosis would allow the development more efficient livestock production systems. The project was implemented using an area-wide integrated pest management strategy including a sterile insect technique (SIT) component. The released sterile male flies originated from a colony from Burkina Faso.

**Methodology/Principal Findings:**

Monitoring the efficacy of the sterile male releases requires the discrimination between wild and sterile male *G*. *p*. *gambiensis* that are sampled in monitoring traps. Before being released, sterile male flies were marked with a fluorescent dye powder. The marking was however not infallible with some sterile flies only slightly marked or some wild flies contaminated with a few dye particles in the monitoring traps. Trapped flies can also be damaged due to predation by ants, making it difficult to discriminate between wild and sterile males using a fluorescence camera and / or a fluorescence microscope. We developed a molecular technique based on the determination of cytochrome oxidase haplotypes of *G*. *p*. *gambiensis* to discriminate between wild and sterile males. DNA was isolated from the head of flies and a portion of the 5’ end of the mitochondrial gene cytochrome oxidase I was amplified to be finally sequenced. Our results indicated that all the sterile males from the Burkina Faso colony displayed the same haplotype and systematically differed from wild male flies trapped in Senegal and Burkina Faso. This allowed 100% discrimination between sterile and wild male *G*. *p*. *gambiensis*.

**Conclusions/Significance:**

This tool might be useful for other tsetse control campaigns with a SIT component in the framework of the Pan-African Tsetse and Trypanosomosis Eradication Campaign (PATTEC) and, more generally, for other vector or insect pest control programs.

## Author Summary

The Government of Senegal has embarked since several years on a project that aims to create a tsetse-free area in the Niayes. The project was implemented using an area-wide integrated pest management (AW-IPM) strategy where the sterile flies used for the sterile insect technique (SIT) component were derived from a colony originating from Burkina Faso. Monitoring the efficacy of the sterile male releases requires the discrimination between wild and sterile males that are sampled in monitoring traps. Before being released, sterile males were marked with a fluorescent dye powder. The marking was however not infallible with some sterile flies only slightly marked or some wild flies contaminated with a few dye particles in the monitoring traps, making it difficult to discriminate between wild and sterile males using a UV camera. We developed a molecular technique based on the cytochrome oxidase gene that efficiently discriminates between wild and sterile males. This tool might be useful for other tsetse control campaigns with a SIT component or for other vector or insect pest control programs.

## Introduction

Tsetse flies (Glossinidae) transmit trypanosomes which cause human African trypanosomosis (HAT) and African animal trypanosomosis (AAT), a debilitating disease of humans (sleeping sickness) and livestock (nagana), respectively [1–4]. The economic cost of AAT in Africa has been estimated at USD 4.75 billion per year [5,6]. For decades, several approaches have been used to manage trypanosomosis, either targeting the parasite using chemotherapy and/or targeting the vector through the use of insecticides. However, less than 2% of the infested area (estimated at around 10 million km²) have been freed of tsetse flies [7]. One of the main reasons for these limited successes has been the reliance on a single control tactic, rather than integrating several control tactics in an area-wide approach [7,8]. There are four methods environmentally and economically acceptable that are currently used in a context of area-wide integrated pest management (AW-IPM) approaches to manage populations of tsetse flies: artificial baits (insecticide-treated traps/targets or ITT), insecticide-treated cattle (ITC), aerial spraying using the sequential aerosol technique (SAT) and the sterile insect technique (SIT) [7,8].

The African Heads of State and Government decided in 2000 to increase efforts to address the tsetse and trypanosomosis problem on the African continent and created the Pan-African Tsetse and Trypanosomiasis Eradication Campaign (PATTEC) [9]. Under this umbrella, the Government of Senegal initiated a tsetse eradication program in the Niayes area that integrated the SIT with other control tactics such as IIT’s and ITC [10,11]. For the SIT component the program used a strain of *Glossina palpalis gambiensis* that was originally colonized at the Centre International de Recherche-Développement sur l’Elevage en zone Subhumide (CIRDES), Bobo-Dioulasso, Burkina Faso. The *G*. *p*. *gambiensis* pupae were mass-reared at the CIRDES and the Slovak Academy of Sciences (SAS), Bratislava, Slovakia and supplemented with excess material from a colony maintained at the FAO/IAEA Insect Pest Control Laboratory, Seibersdorf, Austria and transported as irradiated male pupae by air to Dakar, Senegal [12–14]. The pupae were transferred to an insectary in Dakar for emergence and further processing. The sterile males were marked with a fluorescent dye powder (DayGlo) that was mixed with sand that was put on top of the pupae. Emerging male flies would pick up the powder whilst crawling through the sand, especially in the ptilinum that would later be retracted into the head capsule. The marking of the sterile male flies is required to allow discrimination of sterile from wild flies in the monitoring traps [13] to assess program progress [15]. All flies trapped during the monitoring were transferred to the laboratory and the head capsules examined using a fluorescence camera and / or a fluorescence microscope. In this way, released sterile flies could be distinguished from indigenous flies and the ratio of sterile over wild flies calculated, which is an important parameter for monitoring the efficiency of a SIT campaign [15–17].

However, marking flies with fluorescent dye powder is not infallible, and in some situations some flies can be poorly marked (i.e. with just a few powder particles) or conversely, wild flies in the monitoring traps might become contaminated with a few grains of powder. In addition, tsetse flies in the monitoring traps might be predated upon by ants and lose their head, which makes it difficult to differentiate released sterile flies from wild flies.

Any doubt must be removed on the origin (mass-reared sterile flies or wild flies) of the trapped flies in the field especially in the final eradication phase. A single erroneously classed fly (through poor marking or through contamination) can result in wrong decisions made by the programme managers, i.e. to either continue or stop the eradication process, with potentially large financial losses as a result. Absence of trapped wild flies in continuous monitoring can indeed be considered as an evidence that the population has been eliminated from the target area [18,19].

A more accurate method that removes any doubt on the origin of caught flies would therefore be very useful, and in this paper we present the development of a molecular tool that is based on the mitochondrial gene COI (cytochrome oxidase I) to discriminate sterile from wild males.

## Materials and Methods

### Sampling

Forty eight *G*. *p*. *gambiensis* flies were used to create the reference database of COI sequences i.e. to test the basic hypothesis that the COI sequences of the released *G*. *p*. *gambiensis* flies from the CIRDES colony were different from wild flies in the target areas in Senegal and Burkina Faso. Thirty one of these males originated from the CIRDES colony, seven flies (three males and four females) were collected in Pout (55 km East of Dakar, Senegal) in September 2012 before the release of sterile males had been initiated in this area [12], ten flies were collected in Burkina Faso, of which eight males were from Seguere (50 km North of Bobo-Dioulasso) in December 2013 and two males were from Guinguette (15 km West of Bobo-Dioulasso) in June 2015 (Table 1). The COI sequences of the CIRDES flies were compared with one another and then with those from Pout, Seguere and Guinguette.

**Table 1:** Characteristics of analyzed samples of *G*. *p*. *gambiensis* from various countries and sites.

Country	Site	Operation type	Landscape	Data assignment	Date	Lat	Long	N	Sex
Senegal	Pout	Before release	Niayes	Reference	09/12	14°76	17°05	7	3M+4F
Senegal	Pout	During release	Niayes	Validation	12/14-01/15	14°76	17°05	17	11M+5F+1NI
Senegal	Kayar	During release	Niayes	Validation	07/12	14°89	17°11	3	M
Burkina Faso	CIRDES	Colony	Insectary	Reference	09/12-01/14	11°9	4°17	31	M
Burkina Faso	Seguere	No operation	Gallery forest	Reference	12/13	11°7	4°14	8	M
Burkina Faso	Guinguette	No operation	Gallery forest	Reference	06/15	11°12	4°26	2	M

Date of sampling (month/year), GPS coordinates (Latitude North and Longitude West), N is the subsample sizes, Sex (M for male, F for female, NI for unidentified).

**Fig 1:**
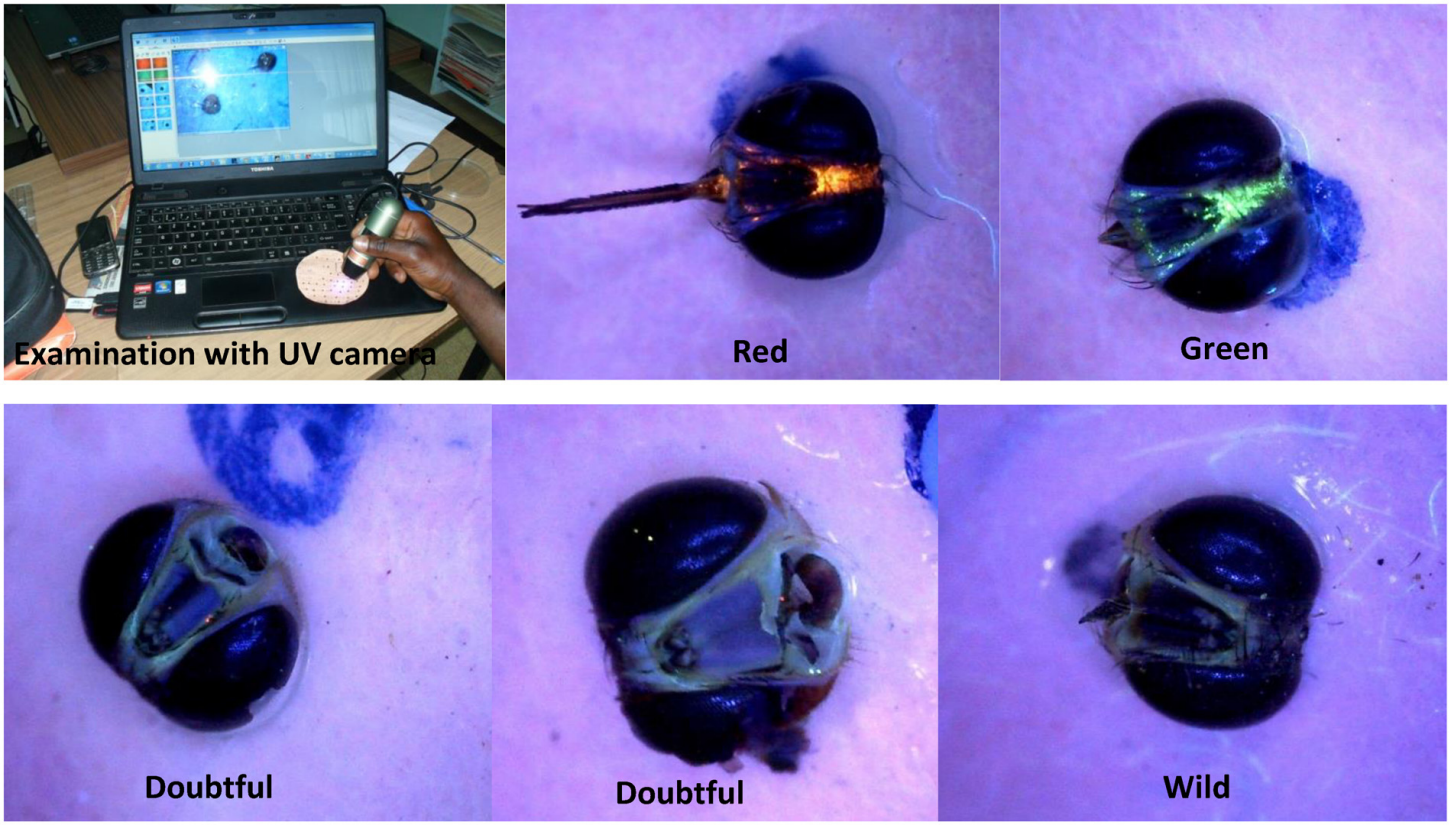
Pictures of (upper left) a UV camera that is used to visualize the fluorescent dye in the head capsules of sterile males, (upper middle and right) red and green fluorescent dye in the head capsule of *Glossina palpalis gambiensis*, (lower left and middle) doubtful marking with only a few powder particles visible, (lower right) head of a wild fly.

The validation of the method was done with twenty flies trapped in the field during the release operations in Senegal: seventeen flies (eleven males, five females and one unidentified sex) collected in Pout in December 2014 and January 2015, and three males collected in Kayar (85 North-East of Dakar, Senegal) in July 2012 (Table 1). Among the seventeen from Pout, ten were analyzed blind at the molecular level while their status (wild or sterile) was known (UV fluorescence reading undoubted) (Fig 1). The remaining seven flies from Pout and the three from Kayar were doubtful using the UV camera (i.e. it was not possible to class them as wild or sterile). The COI sequences of these flies were compared to the reference data i.e. to the sequences of the CIRDES colony flies. Female flies were added in this comparative study because the method used to separate male and female pupae in the mass-rearing insectary is not completely accurate i.e. about 3% female pupae remain in male batch after sex separation and are therefore released together with the sterile males during the release operations [13,14].

Flies from the CIRDES colony were killed in a freezer and kept in 70% alcohol at room temperature (around 25°C). The head of the flies collected in the field was removed and stuck on paper and kept at room temperature. We used heads for the genetic analysis because the discrimination with the fluorescence camera is done on the heads and we used those flies, which gave an unequivocal result with the camera, for discrimination with molecular tools.

### Molecular laboratory methods

DNA was extracted from the head of each tsetse fly using cetyl trimethyl ammonium bromide (CTAB) and chloroform:isoamyl alcohol 24:1 followed by precipitation with isopropanol.

Polymerase chain reaction (PCR) was used to amplify partial sequences of cytochrome oxidase (COI), using the primer pair CI-J-2195/CULR [20]. PCR conditions: 34.9 μl of double distilled water containing 5 μl of 10X PCR buffer, 1 μl of 10 mM dNTP (final concentration 0.2 mM), 1 μl of each 10 μM primer, 6 μl of 25 mM MgCl2 (final concentration 3 mM) were incubated with 0.5U of Taq DNA polymerase (MP Biomedicals) and 1 μl of template DNA. The temperature cycles were: 5 min at 95°C, 40 cycles of 93°C for 1 min, 55°C for 1 min and 72°C for 2 min, then 72°C for 7 min.

PCR products were purified using QIAquick PCR purification kit (Qiagen, Valencia, CA, USA) using the manufacturer’s instructions, then sent for sequencing to GATC biotech. For each PCR product sequenced, forward and reverse sequences were aligned and traces examined using CodonCode Aligner (CodonCodeCorporation). Sequences were aligned and trimmed using Blast in GenBank (https://blast.ncbi.nlm.nih.gov/Blast.cgi?PAGE_TYPE=BlastSearch).

### Ethical statement

The study was conducted in the framework of the tsetse control program in Senegal, implemented by the Division of Veterinary Services, Ministry of Livestock. This project received official approval from the Ministry of Environment of Senegal, under the permit N°0874/MEPN/DE/DEIE/mbf.

## Results

### Molecular reference data

A total of 48 flies were sequenced to set up the molecular reference data. Sequence data for the mitochondrial gene COI (888 nucleotides) were aligned from the 31 individuals of the CIRDES colony. Among these, 29 individuals displayed 100% identity. A substitution (nucleotide T instead of C) was found at position 790 for one individual and one individual showed a sequence of 889 nucleotides including an inserted T after position 822 (Table 2). Of the seven wild individuals that were collected in Pout before the sterile male release operations had started, the sequence of six flies were 100% identical, irrespective of the sex, and one fly (a female) was different from the others by one substitution at position 247 (G instead of A).

**Table 2:** Discrimination between sterile flies from the CIRDES insectary and wild flies from Senegal and Burkina Faso based on a comparison of the COI sequences.

Country	Site	Operation type	Sample size (sex)	Comparison to CIRDES flies
*Molecular reference data*				
Burkina Faso	CIRDES	Colony	31 M	29 are 100% identical
				1 with 1 substitution (T by C at position 790)
				1 with 1 insertion (1T after 822)
	Seguere	No operation	8 M	4 with 1, 2 with 2, 3 with 4 and 1 with 5 substitutions
	Guinguette		2 M	
Senegal	Pout	Before release	7 (3M+4F)	1 with 7 and 6 with 8 substitutions
*Validation of the tool*				
Senegal	Pout	During release	17	13 with 7 to 8 substitutions
			(11M+5F+1NI)	4 are 100% identical
	Kayar	During release	3 M	2 are 100% identical
				1 with 11 substitutions

Sex (M = male, F = female and NI = unidentified sex)

The comparison between sequences of wild flies from Pout and those of sterile flies from the CIRDES colony resulted in substitutions in seven or eight positions (Table 2). The sequences of the wild male flies collected in Seguere (eight flies) and Guinguette (two flies), Burkina Faso, all displayed at least one and up to five substitutions compared to the sequences of the sterile males from the CIRDES colony (Table 2). The detail of the comparison of the COI sequences can be seen in S1 Table.

### Validation of the molecular method

The efficiency of the molecular method was tested using flies collected in the field during the eradication operations in Senegal. A total of twenty flies were caught in the field of which seventeen flies (eleven males, five females and one unidentified) originated from Pout and three male flies from Kayar. Four male individuals trapped in Pout displayed 100% homology with the haplotype of the individuals from the CIRDES colony while thirteen (seven males, five females and one unidentified sex) showed between seven and eight substitutions (almost always the same) along the 888 nucleotides sequence (Table 2). For the three males from Kayar, two individuals displayed 100% identity with the haplotype of the individuals from the CIRDES colony and one differed by eleven substitutions (see S1 Table for the detail of the comparison of COI sequences). This means that the four and two males from Pout and Kayar, respectively were sterile males i.e. from the CIRDES colony while the other individuals were wild flies (Table 2). The results showed that all the ten flies from Pout, of which the status was determined by the UV camera but which were analyzed blindly with the molecular method, were identified correctly (Table 3). The substitute bases and their positions are presented in S1 Dataset.

**Table 3:** Results from visual identification of sterile and wild flies using a UV camera and the molecular method that is based on the sequence of the COI gene.

Collection date	Site	sample	Sex	Camera results	Molecular results
20/07/2012	Kayar	doubtful 22	Male	doubtful	sterile
20/07/2012	Kayar	doubtful 23	Male	doubtful	sterile
20/07/2012	Kayar	doubtful 24	Male	doubtful	wild
19/12/2014	Pout	3 P8-118	Male	doubtful	wild
19/12/2014	Pout	5 P8-118	Male	doubtful	wild
19/12/2014	Pout	7 P8-118	Female	doubtful	wild
30/12/2014	Pout	2 P8-118	Male	doubtful	wild
31/12/2014	Pout	1 P8-118	Female	doubtful	wild
15/01/2015	Pout	3 P8-118	Male	doubtful	wild
16/01/2015	Pout	4 P8-118	unidentified	doubtful	wild
01/01/2015	Pout	1 O8-105	Male	sterile (green)	sterile
30/12/2014	Pout	1 O9-101	Male	sterile (orange)	sterile
02/01/2015	Pout	1 O7-104	Male	sterile (orange)	sterile
19/12/2014	Pout	2 O8-105	Male	sterile (red)	sterile
19/12/2014	Pout	6 P8-118	Female	wild	wild
01/01/2015	Pout	3 P8-118	Female	wild	wild
31/12/2014	Pout	2 O9-101	Male	wild	wild
13/01/2015	Pout	1 P8-118	Male	wild	wild
14/01/2015	Pout	2 P8-118	Female	wild	wild
13/02/1205	Pout	1 P5-106	Male	wild	wild

## Discussion

The aim of this study was to develop a molecular tool to enable discrimination of released sterile males from wild males during an area-wide programme that included an SIT component and that attempted to eradicate a population of *G*. *p*. *gambiensis* from the Niayes in Senegal [12]. This issue has very important implications because the efficiency of the SIT must be assessed during and after the operational releases. At the end of the control activities, the absence of wild flies in the monitoring traps during a reasonable period of time will indicate that the population has been eliminated from the target area and the release operations can be stopped [18,19]. Absence of wild flies in the monitoring traps does not necessarily imply absence of wild flies [21,22]. Indeed, in Senegal, wild flies were recaptured after release of sterile males whereas they were absent in the monitoring traps during eight months [12]. Thus, the efficiency of the trapping system to detect flies seemed to be reduced at very low densities [12]. Probability models are useful to confirm eradication when zero catches are observed for long periods [12]. In the eradication programme against *G*. *austeni* Newstead on the Island of Unguja, Zanzibar, releases of sterile males were thus maintained for six fly generations after trapping the last wild fly before eradication was declared [17]. It is therefore crucial to identify the exact origin (wild or sterile) of trapped flies that are doubtful and difficult to class due to the presence of only a few fluorescent dye powder grains.

Our results showed that the sequence of the mitochondrial gene COI of a series of flies from the CIRDES colony (i.e. used as sterile male flies for release in the Niayes) were mostly identical and differed from the sequence of the COI of wild flies of Pout (in the Niayes). Sequence of the mitochondrial gene COI of wild flies was also mostly identical. These results confirm the basic hypothesis that sterile males that are derived from a colony are almost identical but differed from their wild counterparts. The CIRDES colony is native of Burkina Faso and was established in 1972 from flies collected in the gallery forest of the Guinguette in the Volta River Basin [23]. The Pout area of the Niayes is characterized by artificial vegetation dominated by citrus and mango tree plantations and Euphorbia hedges but without natural surface water [10,24]. The native *G*. *p*. *gambiensis* populations in Pout and the remainder of the Niayes, were highly adapted to artificial vegetation and to strong anthropic pressure [10], as *G*. *p*. *gambiensis* is a fly that normally inhabits Guinean gallery forests [1,25,26]. The ecological differences of the Burkina Faso habitat (origin of the CIRDES flies) and the Pout habitat, and the complete genetic isolation of the Niayes population from the main tsetse belt in West Africa [27,28] may explain the different sequence of the COI gene. Moreover, it has been demonstrated recently that these populations can be considered as different ecotypes [29].

Differences in sequences of the COI gene between *G*. *p*. *gambiensis* flies from the CIRDES colony and their wild counterparts in Burkina Faso were smaller as compared with those between the CIRDES flies and wild flies from the Senegal sites. These reduced differences might be explained by their common genetic background. However, the selection (i.e. genetic drift) pressures to which the CIRDES flies have been subjected for 43 years have resulted in differences (only a single change for four of the samples) that are enough to discriminate between CIRDES colony flies (established in 1972 from wild flies collected in the Guinguette site, Burkina Faso) and wild flies collected in 2015 at the same place (see S1 Dataset for the substitute bases and their positions). Even when only one substitution is present in wild males, its position is clearly different from the one substitution observed in one male from the CIRDES colony and thus sufficient to differentiate all males of the CIRDES colony from wild males in Burkina Faso. The data is very important for any potential future eradication programme in Burkina Faso that incorporates an SIT component and will allow to discriminate flies when identification with the UV camera is doubtful.

Of the twenty *G*. *p*. *gambiensis* flies that were collected in Pout and Kayar during the operational releases of sterile males and that were used for validation of the molecular method, ten were classed as “doubtful” with the UV camera and discrimination between wild or sterile was ambiguous. The molecular method however, could successfully discriminate with 100% accuracy the wild from sterile flies within this group. Indeed, as is shown in Fig 1, some flies had only one or two powder particles on the head and the results of COI sequences highlighted that those flies could be wild or sterile. Similar results were found with *Ceratitis capitata* where the use of a DNA-based marker allowed distinguishing mass-reared flies of the VIENNA 7 and VIENNA 8 strains from wild flies in Western Australia, Guatemala, and Hawaii [30]. The origin of the other ten flies was known but they were analyzed blindly and the molecular results were identical to those obtained with the UV camera. This confirms that the molecular method is highly efficient and can be successfully used to distinguish wild flies from the Niayes area and released sterile flies from the CIRDES colony in the framework of the tsetse eradication program. This tool is proposed only for doubtful samples that can’t be classified using the UV camera. The delay between the collection of the sample and the result from the sequence alignment i.e. to be able to take the right decision at the end of the eradication phase is estimated to maximum 10 days.

The cost of the method is estimated at 12–15 Euro/fly, which includes DNA extraction, PCR, purification of the PCR product and sequencing (corresponding itself to 70–80% of the cost). This cost is not negligible but the expense remains justifiable compared with the large financial losses that might result from wrong decisions due to erroneously classed flies at the end of the eradication phase.

### Conclusion

The tsetse eradication project in the Niayes of Senegal adopted a rolling carpet approach and the entire target area is scheduled to be cleared from *G*. *p*. *gambiensis* by the end of 2017. The preliminary data showed excellent progress in the eradication campaign [31–33]. In this context, the confirmation of the status of “tsetse-free area” in one block before stopping the release operations was very important. The molecular tool developed in this study allowed discrimination with high accuracy between the released sterile males from the CIRDES colony and their wild counterparts. This tool might be useful for other tsetse control campaigns with a SIT component in the framework of the PATTEC. In particular, eight isolated population of *G*. *p*. *gambienis* have been identified recently in West Africa and may be targeted soon by new eradication efforts [28]. More generally, the same tool might be developed for other vector or insect pest control programs.

## Supporting Information

S1 Table Database for the COI sequences of the CIRDES flies and wild flies from Senegal and Burkina Faso and their status from visual identification using a UV camera. (CSV)

S1 Dataset Data for sequence alignments that illustrate the substitutions observed by comparison with the sequence of CIRDES colony flies (CIRDES 9). The substituted bases and positions of these bases are shown in red.
(DOCX)
