# Supplementary material for: A Molecular Method to Discriminate between Mass-Reared Sterile and Wild Tsetse Flies during Eradication Programmes That Have a Sterile Insect Technique Component
Source: PLoS Negl Trop Dis. 2016 Feb 22;10(2):e0004491. doi: 10.1371/journal.pntd.0004491 (PMC4767142; doi:10.1371/journal.pntd.0004491)
Supplement: S1 Dataset — The substituted bases and positions of these bases are shown in red. (DOCX) [file pntd.0004491.s002.docx]

CLUSTAL multiple sequence alignment by Kalign (2.0)

58

CIRDES9 TTGATTTTTTGGTCATCCAGAAGTTTATATTTTGATTCTTCCTGGCTTTGGAATAATTTC

Pout1 TTGATTTTTTGGTCATCCAGAAGTTTATATTTTGATTCTTCCTGGCTTTGGAATAATTTC

Pout6 TTGATTTTTTGGTCATCCAGAAGTTTATATTTTGATTCTTCCTGGCTTTGGAATAATTTC

CIRDES1b TTGATTTTTTGGTCATCCAGAAGTTTATATTTTGATTCTTCCTGGCTTTGGAATAATTTC

CIRDES8b TTGATTTTTTGGTCATCCAGAAGTTTATATTTTGATTCTTCCTGGCTTTGGAATAATTTC

Pout5P8118Male TTGATTTTTTGGTCATCCAGAAGTTTATATTTTGATTCTTCCTGGCTTTGGAATAATTTC

Pout208105Male TTGATTTTTTGGTCATCCAGAAGTTTATATTTTGATTCTTCCTGGCTTTGGAATAATTTC

Pout2P8118Male TTGATTTTTTGGTCATCCAGAAGTTTATATTTTGATTCTTCCTGGCTTTGGAATAATTTC

Kayar22 TTGATTTTTTGGTCATCCAGAAGTTTATATTTTGATTCTTCCTGGCTTTGGAATAATTTC

Kayar24 TTGATTTTTTGGTCATCCAGAAGTTTATATTTTGATTCTTCCTGGCTTTGGAATAAT**A**TC

Bama1 TTGATTTTTTGGTCATCCAGAAGTTTATATTTTGATTCTTCCTGGCTTTGGAATAATTTC

Bama2 TTGATTTTTTGGTCATCCAGAAGTTTATATTTTGATTCTTCCTGGCTTTGGAATAATTTC

Bama3 TTGATTTTTTGGTCATCCAGAAGTTTATATTTTGATTCTTCCTGGCTTTGGAATAATTTC

Bama6 TTGATTTTTTGGTCATCCAGAAGTTTATATTTTGATTCTTCCTGGCTTTGGAATAATTTC

Guiguette1 TTGATTTTTTGGTCATCCAGAAGTTTATATTTTGATTCTTCCTGGCTTTGGAATAATTTC

106 112

CIRDES9 TCATATTATTAGTCAAGAATCTGGAAAAAAAGAAACTTTTGGATCTTTAGGAATAATTTA

Pout1 TCATATTATTAGTCAAGAATCTGGAAAAAAAGAAACTTTTGGATCTTTAGG**G**ATAATTTA

Pout6 TCATATTATTAGTCAAGAATCTGGAAAAAAAGAAACTTTTGGATCTTTAGG**G**ATAATTTA

CIRDES1b TCATATTATTAGTCAAGAATCTGGAAAAAAAGAAACTTTTGGATCTTTAGGAATAATTTA

CIRDES8b TCATATTATTAGTCAAGAATCTGGAAAAAAAGAAACTTTTGGATCTTTAGGAATAATTTA

Pout5P8118Male TCATATTATTAGTCAAGAATCTGGAAAAAAAGAAACTTTTGGATCTTTAGG**G**ATAATTTA

Pout208105Male TCATATTATTAGTCAAGAATCTGGAAAAAAAGAAACTTTTGGATCTTTAGGAATAATTTA

Pout2P8118Male TCATATTATTAGTCAAGAATCTGGAAAAAAAGAAACTTTTGGATCTTTAGG**G**ATAATTTA

Kayar22 TCATATTATTAGTCAAGAATCTGGAAAAAAAGAAACTTTTGGATCTTTAGGAATAATTTA

Kayar24 TCATATTATTAGTCAAGAATCTGGAAAAAAAGAAACTTTTGGATC**C**TTAGGAATAATTTA

Bama1 TCATATTATTAGTCAAGAATCTGGAAAAAAAGAAACTTTTGGATCTTTAGGAATAATTTA

Bama2 TCATATTATTAGTCAAGAATCTGGAAAAAAAGAAACTTTTGGATCTTTAGGAATAATTTA

Bama3 TCATATTATTAGTCAAGAATCTGGAAAAAAAGAAACTTTTGGATCTTTAGGAATAATTTA

Bama6 TCATATTATTAGTCAAGAATCTGGAAAAAAAGAAACTTTTGGATCTTTAGGAATAATTTA

Guiguette1 TCATATTATTAGTCAAGAATCTGGAAAAAAAGAAACTTTTGGATCTTTAGGAATAATTTA

148

CIRDES9 TGCAATATTAGCTATTGGATTATTAGGGTTCATTGTCTGAGCTCATCATATATTTACAGT

Pout1 TGCAATATTAGCTATTGGATTATTAGGGTTCATTGTCTGAGCTCATCATATATTTACAGT

Pout6 TGCAATATTAGCTATTGGATTATTAGGGTTCATTGTCTGAGCTCATCATATATTTACAGT

CIRDES1b TGCAATATTAGCTATTGGATTATTAGGGTTCATTGTCTGAGCTCATCATATATTTACAGT

CIRDES8b TGCAATATTAGCTATTGGATTATTAGGGTTCATTGTCTGAGCTCATCATATATTTACAGT

Pout5P8118Male TGCAATATTAGCTATTGGATTATTAGGGTTCATTGTCTGAGCTCATCATATATTTACAGT

Pout208105Male TGCAATATTAGCTATTGGATTATTAGGGTTCATTGTCTGAGCTCATCATATATTTACAGT

Pout2P8118Male TGCAATATTAGCTATTGGATTATTAGGGTTCATTGTCTGAGCTCATCATATATTTACAGT

Kayar22 TGCAATATTAGCTATTGGATTATTAGGGTTCATTGTCTGAGCTCATCATATATTTACAGT

Kayar24 TGCAATATTAGCTATTGGATTATTAGGGTTCATTGTCTGAGCTCATCATATATTTACAGT

Bama1 TGCAATATTAGCTATTGGATTATTAGG**A**TTCATTGTCTGAGCTCATCATATATTTACAGT

Bama2 TGCAATATTAGCTATTGGATTATTAGG**A**TTCATTGTCTGAGCTCATCATATATTTACAGT

Bama3 TGCAATATTAGCTATTGGATTATTAGGGTTCATTGTCTGAGCTCATCATATATTTACAGT

Bama6 TGCAATATTAGCTATTGGATTGTTAGG**A**TTCATTGTCTGAGCTCATCACATATTTACAGT

Guiguette1 TGCAATATTAGCTATTGGATTATTAGG**A**TTCATTGTCTGAGCTCATCATATATTTACAGT

CIRDES9 AGGTATAGACGTAGATACCCGAGCCTATTTTACTTCTGCAACAATAATTATTGCTGTTCC

Pout1 AGGTATAGACGTAGATACCCGAGCCTATTTTACTTCTGCAACAATAATTATTGCTGTTCC

Pout6 AGGTATAGACGTAGATACCCGAGCCTATTTTACTTCTGCAACAATAATTATTGCTGTTCC

CIRDES1b AGGTATAGACGTAGATACCCGAGCCTATTTTACTTCTGCAACAATAATTATTGCTGTTCC

CIRDES8b AGGTATAGACGTAGATACCCGAGCCTATTTTACTTCTGCAACAATAATTATTGCTGTTCC

Pout5P8118Male AGGTATAGACGTAGATACCCGAGCCTATTTTACTTCTGCAACAATAATTATTGCTGTTCC

Pout208105Male AGGTATAGACGTAGATACCCGAGCCTATTTTACTTCTGCAACAATAATTATTGCTGTTCC

Pout2P8118Male AGGTATAGACGTAGATACCCGAGCCTATTTTACTTCTGCAACAATAATTATTGCTGTTCC

Kayar22 AGGTATAGACGTAGATACCCGAGCCTATTTTACTTCTGCAACAATAATTATTGCTGTTCC

Kayar24 AGGTATAGACGTAGATACCCGAGCCTATTTTACTTCTGCAACAATAATTATTGCTGTTCC

Bama1 AGGTATAGACGTAGATACCCGAGCCTATTTTACTTCTGCAACAATAATTATTGCTGTTCC

Bama2 AGGTATAGACGTAGATACCCGAGCCTATTTTACTTCTGCAACAATAATTATTGCTGTTCC

Bama3 AGGTATAGACGTAGATACCCGAGCCTATTTTACTTCTGCAACAATAATTATTGCTGTTCC

Bama6 AGGTATAGACGTAGATACCCGAGCCTATTTTACTTCTGCAACAATAATTATTGCTGTTCC

Guiguette1 AGGTATAGACGTAGATACCCGAGCCTATTTTACTTCTGCAACAATAATTATTGCTGTTCC

247 269 283

CIRDES9 AACTGGGATTAAAATTTTTAGTTGATTAGCTACTCTTCACGGGACTCAAATTTCTTACTC

Pout1 AACTGG**A**ATTAAAATTTTTAGTTGATTAGCTACTCTTCACGG**A**ACTCAAATTTCTTACTC

Pout6 AACTGGGATTAAAATTTTTAGTTGATTAGCTACTCTTCACGG**A**ACTCAAATTTCTTACTC

CIRDES1b AACTGGGATTAAAATTTTTAGTTGATTAGCTACTCTTCACGGGACTCAAATTTCTTACTC

CIRDES8b AACTGGGATTAAAATTTTTAGTTGATTAGCTACTCTTCACGGGACTCAAATTTCTTACTC

Pout5P8118Male AACTGG**A**ATTAAAATTTTTAGTTGATTAGCTACTCTTCACGG**A**ACTCAAATTTCTTACTC

Pout208105Male AACTGGGATTAAAATTTTTAGTTGATTAGCTACTCTTCACGGGACTCAAATTTCTTACTC

Pout2P8118Male AACTGGGATTAAAATTTTTAGTTGATTAGCTACTCTTCACGG**A**ACTCAAATTTCTTACTC

Kayar22 AACTGGGATTAAAATTTTTAGTTGATTAGCTACTCTTCACGGGACTCAAATTTCTTACTC

Kayar24 AACTGGGATTAAAATTTTTAGTTGATTA**C**CTACTCTTCACGGGACTCAAATTTCTTACTC

Bama1 AACTGGGATTAAAATTTTTAGTTGATTAGCTACTCTTCACGGGACTCAAATTTCTTACTC

Bama2 AACTGG**A**ATTAAAATTTTTAGTTGATTAGCTACTCTTCACGGGACTCAAATTTCTTACTC

Bama3 AACTGG**A**ATTAAAATTTTTAGTTGATTAGCTACTCTTCACGGGACTCAAATTTCTTACTC

Bama6 AACTGGGATTAAAATTTTTAGTTGATTAGCTACTCTTCACGGGACTCAAATTTCTTACTC

Guiguette1 AACTGGGATTAAAATTTTTAGTTGATTAGCTACTCTTCACGGGACTCAAATTTCTTACTC

CIRDES9 TCCGGCTATTTTATGAGCCCTTGGGTTTATTTTTCTATTTACTGTAGGAGGTTTAACAGG

Pout1 TCCGGCTATTTTATGAGCCCTTGGGTTTATTTTTCTATTTACTGTAGGAGGTTTAACAGG

Pout6 TCCGGCTATTTTATGAGCCCTTGGGTTTATTTTTCTATTTACTGTAGGAGGTTTAACAGG

CIRDES1b TCCGGCTATTTTATGAGCCCTTGGGTTTATTTTTCTATTTACTGTAGGAGGTTTAACAGG

CIRDES8b TCCGGCTATTTTATGAGCCCTTGGGTTTATTTTTCTATTTACTGTAGGAGGTTTAACAGG

Pout5P8118Male TCCGGCTATTTTATGAGCCCTTGGGTTTATTTTTCTATTTACTGTAGGAGGTTTAACAGG

Pout208105Male TCCGGCTATTTTATGAGCCCTTGGGTTTATTTTTCTATTTACTGTAGGAGGTTTAACAGG

Pout2P8118Male TCCGGCTATTTTATGAGCCCTTGGGTTTATTTTTCTATTTACTGTAGGAGGTTTAACAGG

Kayar22 TCCGGCTATTTTATGAGCCCTTGGGTTTATTTTTCTATTTACTGTAGGAGGTTTAACAGG

Kayar24 TCCGGCTATTTTATGAGCCCTTGGGTTTATTTTTCTATTTACTGTAGGAGGTTTAACAGG

Bama1 TCCGGCTATTTTATGAGCCCTTGGGTTTATTTTTCTATTTACTGTAGGAGGTTTAACAGG

Bama2 TCCGGCTATTTTATGAGCCCTTGGGTTTATTTTTCTATTTACTGTAGGAGGTTTAACAGG

Bama3 TCCGGCTATTTTATGAGCCCTTGGGTTTATTTTTCTATTTACTGTAGGAGGTTTAACAGG

Bama6 TCCGGCTATTTTATGAGCCCTTGGGTTTATTTTTCTATTTACTGTAGGAGGTTTAACAGG

Guiguette1 TCCGGCTATTTTATGAGCCCTTGGGTTTATTTTTCTATTTACTGTAGGAGGTTTAACAGG

CIRDES9 AGTAGTTTTAGCTAATTCTTCAGTTGACATCATTCTCCATGATACCTATTATGTTGTTGC

Pout1 AGTAGTTTTAGCTAATTCTTCAGTTGACATCATTCTCCATGATACCTATTATGTTGTTGC

Pout6 AGTAGTTTTAGCTAATTCTTCAGTTGACATCATTCTCCATGATACCTATTATGTTGTTGC

CIRDES1b AGTAGTTTTAGCTAATTCTTCAGTTGACATCATTCTCCATGATACCTATTATGTTGTTGC

CIRDES8b AGTAGTTTTAGCTAATTCTTCAGTTGACATCATTCTCCATGATACCTATTATGTTGTTGC

Pout5P8118Male AGTAGTTTTAGCTAATTCTTCAGTTGACATCATTCTCCATGATACCTATTATGTTGTTGC

Pout208105Male AGTAGTTTTAGCTAATTCTTCAGTTGACATCATTCTCCATGATACCTATTATGTTGTTGC

Pout2P8118Male AGTAGTTTTAGCTAATTCTTCAGTTGACATCATTCTCCATGATACCTATTATGTTGTTGC

Kayar22 AGTAGTTTTAGCTAATTCTTCAGTTGACATCATTCTCCATGATACCTATTATGTTGTTGC

Kayar24 AGTAGTTTTAGCTAATTCTTCAGTTGACATCATTCTCCATGATACCTATTATGTTGTTGC

Bama1 AGTAGTTTTAGCTAATTCTTCAGTTGACATCATTCTCCATGATACCTATTATGTTGTTGC

Bama2 AGTAGTTTTAGCTAATTCTTCAGTTGACATCATTCTCCATGATACCTATTATGTTGTTGC

Bama3 AGTAGTTTTAGCTAATTCTTCAGTTGACATCATTCTCCATGATACCTATTATGTTGTTGC

Bama6 AGTAGTTTTAGCTAATTCTTCAGTTGACATCATTCTCCATGATACCTATTATGTTGTTGC

Guiguette1 AGTAGTTTTAGCTAATTCTTCAGTTGACATCATTCTCCATGATACCTATTATGTTGTTGC

CIRDES9 TCATTTTCACTATGTTTTATCAATAGGAGCAGTATTTGCTATTATAGCAGGCTTTATCCA

Pout1 TCATTTTCACTATGTTTTATCAATAGGAGCAGTATTTGCTATTATAGCAGGCTTTATCCA

Pout6 TCATTTTCACTATGTTTTATCAATAGGAGCAGTATTTGCTATTATAGCAGGCTTTATCCA

CIRDES1b TCATTTTCACTATGTTTTATCAATAGGAGCAGTATTTGCTATTATAGCAGGCTTTATCCA

CIRDES8b TCATTTTCACTATGTTTTATCAATAGGAGCAGTATTTGCTATTATAGCAGGCTTTATCCA

Pout5P8118Male TCATTTTCACTATGTTTTATCAATAGGAGCAGTATTTGCTATTATAGCAGGCTTTATCCA

Pout208105Male TCATTTTCACTATGTTTTATCAATAGGAGCAGTATTTGCTATTATAGCAGGCTTTATCCA

Pout2P8118Male TCATTTTCACTATGTTTTATCAATAGGAGCAGTATTTGCTATTATAGCAGGCTTTATCCA

Kayar22 TCATTTTCACTATGTTTTATCAATAGGAGCAGTATTTGCTATTATAGCAGGCTTTATCCA

Kayar24 TCATTTTCACTATGTTTTATCAATAGGAGCAGTATTTGCTATTATAGCAGGCTTTATCCA

Bama1 TCATTTTCACTATGTTTTATCAATAGGAGCAGTATTTGCTATTATAGCAGGCTTTATCCA

Bama2 TCATTTTCACTATGTTTTATCAATAGGAGCAGTATTTGCTATTATAGCAGGCTTTATCCA

Bama3 TCATTTTCACTATGTTTTATCAATAGGAGCAGTATTTGCTATTATAGCAGGCTTTATCCA

Bama6 TCATTTTCACTATGTTTTATCAATAGGAGCAGTATTTGCTATTATAGCAGGCTTTATCCA

Guiguette1 TCATTTTCACTATGTTTTATCAATAGGAGCAGTATTTGCTATTATAGCAGGCTTTATCCA

511

CIRDES9 TTGATATCCTTTATTTACAGGTTTAACTATAAATACATCAATATTAAAAAGTCAATTTAT

Pout1 TTGATATCCTTTATTTACAGGTTTAACTAT**G**AATACATCAATATTAAAAAGTCAATTTAT

Pout6 TTGATATCCTTTATTTACAGGTTTAACTAT**G**AATACATCAATATTAAAAAGTCAATTTAT

CIRDES1b TTGATATCCTTTATTTACAGGTTTAACTATAAATACATCAATATTAAAAAGTCAATTTAT

CIRDES8b TTGATATCCTTTATTTACAGGTTTAACTATAAATACATCAATATTAAAAAGTCAATTTAT

Pout5P8118Male TTGATATCCTTTATTTACAGGTTTAACTAT**G**AATACATCAATATTAAAAAGTCAATTTAT

Pout208105Male TTGATATCCTTTATTTACAGGTTTAACTATAAATACATCAATATTAAAAAGTCAATTTAT

Pout2P8118Male TTGATATCCTTTATTTACAGGTTTAACTAT**G**AATACATCAATATTAAAAAGTCAATTTAT

Kayar22 TTGATATCCTTTATTTACAGGTTTAACTATAAATACATCAATATTAAAAAGTCAATTTAT

Kayar24 TTGATATCCTTTATTTACAGGTTTAACTATAAATACATCAATATTAAAAAGTCAATTTAT

Bama1 TTGATATCCTTTATTTACAGGTTTAACTATAAATACATCAATATTAAAAAGTCAATTTAT

Bama2 TTGATATCCTTTATTTACAGGTTTAACTATAAATACATCAATATTAAAAAGTCAATTTAT

Bama3 TTGATATCCTTTATTTACAGGTTTAACTATAAATACATCAATATTAAAAAGTCAATTTAT

Bama6 TTGATATCCTTTATTTACAGGTTTAACTATAAATACATCAATATTAAAAAGTCAATTTAT

Guiguette1 TTGATATCCTTTATTTACAGGTTTAACTATAAATACATCAATATTAAAAAGTCAATTTAT

CIRDES9 AGTAATATTTATCGGAGTAAATTTAACATTTTTTCCTCAACATTTTTTAGGTTTAGCAGG

Pout1 AGTAATATTTATCGGAGTAAATTTAACATTTTTTCCTCAACATTTTTTAGGTTTAGCAGG

Pout6 AGTAATATTTATCGGAGTAAATTTAACATTTTTTCCTCAACATTTTTTAGGTTTAGCAGG

CIRDES1b AGTAATATTTATCGGAGTAAATTTAACATTTTTTCCTCAACATTTTTTAGGTTTAGCAGG

CIRDES8b AGTAATATTTATCGGAGTAAATTTAACATTTTTTCCTCAACATTTTTTAGGTTTAGCAGG

Pout5P8118Male AGTAATATTTATCGGAGTAAATTTAACATTTTTTCCTCAACATTTTTTAGGTTTAGCAGG

Pout208105Male AGTAATATTTATCGGAGTAAATTTAACATTTTTTCCTCAACATTTTTTAGGTTTAGCAGG

Pout2P8118Male AGTAATATTTATCGGAGTAAATTTAACATTTTTTCCTCAACATTTTTTAGGTTTAGCAGG

Kayar22 AGTAATATTTATCGGAGTAAATTTAACATTTTTTCCTCAACATTTTTTAGGTTTAGCAGG

Kayar24 AGTAATATTTATCGGAGTAAATTTAACATTTTTTCCTCAACATTTTTTAGGTTTAGCAGG

Bama1 AGTAATATTTATCGGAGTAAATTTAACATTTTTTCCTCAACATTTTTTAGGTTTAGCAGG

Bama2 AGTAATATTTATCGGAGTAAATTTAACATTTTTTCCTCAACATTTTTTAGGTTTAGCAGG

Bama3 AGTAATATTTATCGGAGTAAATTTAACATTTTTTCCTCAACATTTTTTAGGTTTAGCAGG

Bama6 AGTAATATTTATCGGAGTAAATTTAACATTTTTTCCTCAACATTTTTTAGGTTTAGCAGG

Guiguette1 AGTAATATTTATCGGAGTAAATTTAACATTTTTTCCTCAACATTTTTTAGGTTTAGCAGG

CIRDES9 TATACCTCGTCGTTATTCTGATTACCCAGATGCTTATACAACTTGAAACGTAATTTCAAC

Pout1 TATACCTCGTCGTTATTCTGATTACCCAGATGCTTATACAACTTGAAACGTAATTTCAAC

Pout6 TATACCTCGTCGTTATTCTGATTACCCAGATGCTTATACAACTTGAAACGTAATTTCAAC

CIRDES1b TATACCTCGTCGTTATTCTGATTACCCAGATGCTTATACAACTTGAAACGTAATTTCAAC

CIRDES8b TATACCTCGTCGTTATTCTGATTACCCAGATGCTTATACAACTTGAAACGTAATTTCAAC

Pout5P8118Male TATACCTCGTCGTTATTCTGATTACCCAGATGCTTATACAACTTGAAACGTAATTTCAAC

Pout208105Male TATACCTCGTCGTTATTCTGATTACCCAGATGCTTATACAACTTGAAACGTAATTTCAAC

Pout2P8118Male TATACCTCGTCGTTATTCTGATTACCCAGATGCTTATACAACTTGAAACGTAATTTCAAC

Kayar22 TATACCTCGTCGTTATTCTGATTACCCAGATGCTTATACAACTTGAAACGTAATTTCAAC

Kayar24 TATACCTCGTCGTTATTCTGATTACCCAGATGCTTATACAACTTGAAACGTAATTTCAAC

Bama1 TATACCTCGTCGTTATTCTGATTACCCAGATGCTTATACAACTTGAAACGTAATTTCAAC

Bama2 TATACCTCGTCGTTATTCTGATTACCCAGATGCTTATACAACTTGAAACGTAATTTCAAC

Bama3 TATACCTCGTCGTTATTCTGATTACCCAGATGCTTATACAACTTGAAACGTAATTTCAAC

Bama6 TATACCTCGTCGTTATTCTGATTACCCAGATGCTTATACAACTTGAAACGTAATTTCAAC

Guiguette1 TATACCTCGTCGTTATTCTGATTACCCAGATGCTTATACAACTTGAAACGTAATTTCAAC

673 676 685 688 712

CIRDES9 AATTGGATCAACAATTTCTTTATTGGGAATTATTTTTTTCTTTTTCATTATCTGAGAAAG

Pout1 AATTGGATCAACAATTTCTTTATT**A**GGAATTATTTTTTTCTTTTTCATTAT**T**TGAGAAAG

Pout6 AATTGGATCAACAATTTCTTTATT**A**GGAATTATTTTTTTCTTTTTCATTAT**T**TGAGAAAG

CIRDES1b AATTGGATCAACAATTTCTTTATTGGGAATTATTTTTTTCTTTTTCATTATCTGAGAAAG

CIRDES8b AATTGGATCAACAATTTCTTTATTGGGAATTATTTTTTTCTTTTTCATTATCTGAGAAAG

Pout5P8118Male AATTGGATCAACAATTTCTTTATT**A**GGAATTATTTTTTTCTTTTTCATTAT**T**TGAGAAAG

Pout208105Male AATTGGATCAACAATTTCTTTATTGGGAATTATTTTTTTCTTTTTCATTATCTGAGAAAG

Pout2P8118Male AATTGGATCAACAATTTCTTTATT**A**GGAATTATTTTTTTCTTTTTCATTAT**T**TGAGAAAG

Kayar22 AATTGGATCAACAATTTCTTTATTGGGAATTATTTTTTTCTTTTTCATTATCTGAGAAAG

Kayar24 AATTGGATCAACAATTTCTTTATTGGGAATTATTTTTTTCTTTTTCATTATCTGAGAAAG

Bama1 AATTGGATCAACAATTTCTTTATTGGGAATTATTTTTTTCTTTTTCATTATCTGAGAAAG

Bama2 AATTGGATCAACAATTTCTTTATTGGGAATTATTTTTTTCTTTTTCATTATCTGAGAAAG

Bama3 AATTGGATCAACAAT**C**TCTTTATTGGG**G**ATTATTTTTTTCTTTTTCATTATCTGAGAAAG

Bama6 AATTGGATCAAC**G**ATTTCTTTATTGGGAATTATTTTTTTCTTTTTCATTATCTGAGAAAG

Guiguette1 AATTGGATCAAC**G**ATTTCTTTATTGGGAATTATTTTTTTCTTTTTCATTATCTGAGAAAG

CIRDES9 ATTAGTTAGCCAACGAAAAGTTATTTTTCCTATTCAATTAAATTCTTCAATTGAATGACT

Pout1 ATTAGTTAGCCAACGAAAAGTTATTTTTCCTATTCAATTAAATTCTTCAATTGAATGACT

Pout6 ATTAGTTAGCCAACGAAAAGTTATTTTTCCTATTCAATTAAATTCTTCAATTGAATGACT

CIRDES1b ATTAGTTAGCCAACGAAAAGTTATTTTTCCTATTCAATTAAATTCTTCAATTGAATGACT

CIRDES8b ATTAGTTAGCCAACGAAAAGTTATTTTTCCTATTCAATTANATTCTTCAATTGAATGACT

Pout5P8118Male ATTAGTTAGCCAACGAAAAGTTATTTTTCCTATTCAATTAAATTCTTCAATTGAATGACT

Pout208105Male ATTAGTTAGCCAACGAAAAGTTATTTTTCCTATTCAATTAAATTCTTCAATTGAATGACT

Pout2P8118Male ATTAGTTAGCCAACGAAAAGTTATTTTTCCTATTCAATTAAATTCTTCAATTGAATGACT

Kayar22 ATTAGTTAGCCAACGAAAAGTTATTTTTCCTATTCAATTAAATTCTTCAATTGAATGACT

Kayar24 ATTAGTTAGCCAACGAAAAGTTATTTTTCCTATTCAATTAAATTCTTCAATTGAATGACT

Bama1 ATTAGTTAGCCAACGAAAAGTTATTTTTCCTATTCAATTAAATTCTTCAATTGAATGACT

Bama2 ATTAGTTAGCCAACGAAAAGTTATTTTTCCTATTCAATTAAATTCTTCAATTGAATGACT

Bama3 ATTAGTTAGCCAACGAAAAGTTATTTTTCCTATTCAATTAAATTCTTCAATTGAATGACT

Bama6 ATTAGTTAGCCAACGAAAAGTTATTTTTCCTATTCAATTAAATTCTTCAATTGAATGACT

Guiguette1 ATTAGTTAGCCAACGAAAAGTTATTTTTCCTATTCAATTAAATTCTTCAATTGAATGACT

790 805 823

CIRDES9 ACAAAATACCCCTCCTTCTGAACATAGTTATTCCGAATTACC-TTTATTAACTAATTAAC

Pout1 ACAAAATAC**T**CCTCCTTCTGAACATAGTTATTCCGAATTACC-**C**TTATTAACTAATTAAC

Pout6 ACAAAATAC**T**CCTCCTTCTGAACATAGTTATTCCGAATTACC-**C**TTATTAACTAATTAAC

CIRDES1b ACAAAATAC**T**CCTCCTTCTGAACATAGTTATTCCGAATTACC-TTTATTAACTAATTAAC

CIRDES8b ACAAAATACCCCTCCTTCTGAACATAGTTATTCCGAATTACC**T**TTTATTAACTNATTAAC

Pout5P8118Male ACAAAATAC**T**CCTCCTTCTGAACATAGTTATTCCGAATTACC-**C**TTATTAACTAATTAAC

Pout208105Male ACAAAATACCCCTCCTTCTGAACATAGTTATTCCGAATTACC-TTTATTAACTAATTAAC

Pout2P8118Male ACAAAATAC**T**CCTCCTTCTGAACATAGTTATTCCGAATTACC-**C**TTATTAACTAATTAAC

Kayar22 ACAAAATACCCCTCCTTCTGAACATAGTTATTCCGAATTACC-TTTATTAACTAATTAAC

Kayar24 ACAAAATACCCCTCCTTCTGAACATAGTTATTCCGAATTACC-TTTATTAACTAATTAAC

Bama1 ACAAAATACCCCTCCTTCTGAACATAGTTATTCCGAATTACC-TTTATTAACTAATTAAC

Bama2 ACAAAATACCCCTCCTTCTGAACATAGTTATTCCGAATTACC-TTTATTAACTAATTAAC

Bama3 ACAAAATACCCCTCCTTCTGAACA**C**AGTTATTCCGAATTACC-TTTATTAACTAATTAAC

Bama6 ACAAAATACCCCTCCTTCTGAACATAGTTATTCCGAATTACC-TTTATTAACTAATTAAC

Guiguette1 ACAAAATACCCCTCCTTCTGAACATAGTTATTCCGAATTACC-TTTATTAACTAATTAAC

CIRDES9 TAGTTATTAATTCTAATATGGCAGATTAGTGCAATGAATTTAAGCTTCA

Pout1 TAGTTATTAATTCTAATATGGCAGATTAGTGCAATGAATTTAAGCTTCA

Pout6 TAGTTATTAATTCTAATATGGCAGATTAGTGCAATGAATTTAAGCTTCA

CIRDES1b TAGTTATTAATTCTAATATGGCAGATTAGTGCAATGAATTTAAGCTTCA

CIRDES8b TANTTATTAATTCTAATATGGCAGATTAGTGCAATGAATTTAAGCTTCA

Pout5P8118Male TAGTTATTAATTCTAATATGGCAGATTAGTGCAATGAATTTAAGCTTCA

Pout208105Male TAGTTATTAATTCTAATATGGCAGATTAGTGCAATGAATTTAAGCTTCA

Pout2P8118Male TAGTTATTAATTCTAATATGGCAGATTAGTGCAATGAATTTAAGCTTCA

Kayar22 TAGTTATTAATTCTAATATGGCAGATTAGTGCAATGAATTTAAGCTTCA

Kayar24 TAGTTATTAATTCTAATATGGCAGATTAGTGCAATGAATTTAAGCTTCA

Bama1 TAGTTATTAATTCTAATATGGCAGATTAGTGCAATGAATTTAAGCTTCA

Bama2 TAGTTATTAATTCTAATATGGCAGATTAGTGCAATGAATTTAAGCTTCA

Bama3 TAGTTATTAATTCTAATATGGCAGATTAGTGCAATGAATTTAAGCTTCA

Bama6 TAGTTATTAATTCTAATATGGCAGATTAGTGCAATGAATTTAAGCTTCA

Guiguette1 TAGTTATTAATTCTAATATGGCAGATTAGTGCAATGAATTTAAGCTTCA
